# Abnormal functional connectivity in the habenula is associated with subjective hyperarousal state in chronic insomnia disorder

**DOI:** 10.3389/fneur.2023.1119595

**Published:** 2023-07-31

**Authors:** Liang Gong, Fang Cheng, Xue Li, Zhiqi Wang, Shuo Wang, Ronghua Xu, Bei Zhang, Chunhua Xi

**Affiliations:** ^1^Department of Neurology, Chengdu Second People’s Hospital, Chengdu, Sichuan, China; ^2^Department of Neurology, The Third Affiliated Hospital of Anhui Medical University, Hefei, Anhui, China

**Keywords:** insomnia disorder, habenula, hyperarousal, functional connectivity, caudate nucleus

## Abstract

**Background:**

The hyperarousal process model plays a central role in the physiology of chronic insomnia disorder (CID). Recent evidence has demonstrated that the habenula is involved in the arousal and sleep–wake cycle. However, whether the intrinsic habenular functional network contributes to the underlying mechanism of CID and its relationship to the arousal state in CID remains unclear.

**Methods:**

This single-centered study included 34 patients with subjective CID and 22 matched good sleep control (GSC), and underwent a series of neuropsychological tests and resting-state functional magnetic resonance imaging scans. The habenular functional network was assessed using seed-based functional connectivity (FC) analysis. The subjective arousal state was evaluated with the hyperarousal scale (HAS). Alterations in the habenular FC network and their clinical significance in patients with CID were explored.

**Results:**

Compared with the GSC group, the CID group showed decreased habenular FC in the left caudate nucleus and right inferior parietal lobule and increased FC in the right habenula, bilateral calcarine cortex, and posterior cingulate cortex. The decreased FC between the left habenula and caudate nucleus was associated with an increased arousal state in the CID group.

**Conclusion:**

The present results provide evidence for a dysfunctional habenular network in patients with CID. These findings extend our understanding of the neuropathological mechanisms underlying the hyperarousal model in chronic insomnia.

## 1. Introduction

Insomnia is a frequent clinical disorder characterized by difficulty falling or staying asleep and is accompanied by symptoms, including irritability or exhaustion during the day. Insomnia affects approximately 10–20% of people, with approximately half of those suffering from a chronic course (1), such as chronic insomnia disorder (CID). CID is highly comorbid with and linked to mental health disorders, such as anxiety and depression (2). Despite the high morbidity rate and widespread socioeconomic impact of CID, its underlying neuropathological mechanisms remain unclear. Currently, the hyperarousal process model is considered to have a circuital role in the physiological and pathological aspects of insomnia (3). However, the detailed neurobiological basis of hyperarousal remains unclear (4).

Neuroimaging data recognizes the abnormal brain function and structure in patients with insomnia (5, 6), especially using functional connectivity (FC) approaches (7). Patients with insomnia present functional and structural alterations in multiple brain regions, including the hippocampus, amygdala, insula, prefrontal cortex, anterior cingulate cortex, pineal gland, arousal system (hypothalamus), salience network, reward network, and default mode network (8, 9). In addition, the habenula has been considered to play an important role in sleep and circadian rhythms (10). However, whether the functional alterations to the habenula play a role in insomnia remains unelucidated.

The habenular nuclei along with the pineal gland constitute the epithalamus and are evolutionarily conserved structures that show phylogenetic conservation from fish to humans (11). Melatonin mediates physiological effects by activating two G protein-coupled receptors and is secreted from the pineal gland. These receptors are expressed in several brain areas and information is transmitted from the forebrain to the midbrain, including the habenular complex, and modulate a plethora of behaviors including mood, sleep, and pain. The excitability of medial-lateral habenula neurons is enhanced by melatonin receptor activation (12). However, the habenula has been shown to regulate the monoaminergic systems in the central nervous system and is activated by negatively valued events (13). Previous studies have found that the habenula is involved in regulating negative emotion and motivation, reward processing, mediating stress responses, and sleep–wake cycles (10, 14). Dopamine (DA) is a key neurotransmitter involved in multiple physiological functions, including modulation of affective and emotional states, motor control, and reward mechanisms (15). Previous evidence has shown that the lateral habenula is linked to mood and reward regulation through modulation of the DA system (16). For example, the ventral pallidum DRD3+ neuronal populations projecting to the lateral habenula (LHb) display activity during drug-seeking behavior, and selective suppression of elevated activity or DRD3 signaling in the LHb-projecting population reduces drug-seeking behavior (17). Animal studies have also shown that damage to the habenular nucleus leads to hyperkinetic and impulsive behavior and biochemical mechanisms are thought to affect DA level in the mesolimbic system (18). However, the habenula’s function is disrupted in patients with major depression (19), and hyperactivation of the habenula might contribute to both depression and sleep disturbance (20).

Along with the advancement of neuroimaging, researchers have attempted to evaluate habenular function using resting-state functional magnetic resonance imaging (rs-fMRI)-based FC analysis (21). Patients with chronic pain have been found to have altered habenular FC (22) and subclinical depression (23). Previous research has focused on the role of habenula function, with particular attention to its resting-state connectivity and potential as an early biomarker for neuropsychiatric disorders. Currently, no study has explored whether the habenular function is abnormal in patients with CID, although the habenula plays an important role in sleep–wake cycles.

Therefore, this novel study aimed to investigate the potential functional alterations of the habenula in patients with CID and their relationship with the clinical and neuropsychological features of insomnia. Given the role of the habenula in regulating reward, sleep–wake cycles, and emotions, we hypothesized that abnormal habenular FC could be found in reward- and emotion-related networks. Furthermore, we speculated that altered habenular function would be associated with mental symptoms and features of insomnia in patients with CID.

## 2. Materials and methods

### 2.1. Participants

This study was approved by the ethical committee of the Institutional Review Board of the Third Affiliated Hospital of Anhui Medical University (approval number 2019–010-01), and was conducted in accordance with the Declaration of Helsinki. We obtained written informed consent from all patients.

We enrolled all patients in the outpatient department of the Third Affiliated Hospital of Anhui Medical University. In total, 34 patients with subjective CID and 22 age-, education-, and sex-matched GSC participants were included in this study and underwent a series of neuropsychological tests and resting-state fMRI scans. All participants were of Han Chinese descent and right-handed. Four participants (two CID and two GSC) were excluded due to excessive head motion artifacts (>2 mm or 2°; see the preprocessing of fMRI data for details); thus, 32 CID and 20 GSC participants were included in the final analysis.

In the CID group, patients were required to meet the diagnostic criteria for CID outlined in the International Classification of Sleep Disorders, third version (24). Additional inclusion criteria for the CID group were as follows: (1) no intake of any hypnotic or antidepressant medication 2 weeks before the neuropsychological test and MRI scan; and (2) age between 18 and 55 years with age at onset <50 years. The exclusion criteria were as follows: (1) a history of other neuropsychiatric disorders; (2) a history of substance abuse (caffeine, nicotine, alcohol); and (3) contraindications to MRI. Subjects with GSC were required to meet the following criteria: (1) good sleep, mood, and normal cognitive function; (2) no history of neurological or psychiatric disease, seizures, head injury, stroke, or transient ischemic attack; (3) no caffeine, drug, or alcohol abuse; and (4) no brain lesions found on a regular T2-weighted MRI scan.

### 2.2. Neuropsychological test

The Pittsburgh Sleep Quality Index (PSQI) and insomnia severity index (ISI) scale were used to assess subjective sleep quality and insomnia severity, respectively (25, 26). The hyperarousal scale (HAS) was employed to assess the subjective arousal state (27). Epworth sleepiness scale (ESS) was used to measure daytime sleepiness (28). Zung’s self-rating depression scale (SDS) and Zung’s self-rating anxiety scale (SAS) were used for depression and anxiety evaluation, respectively. The mini-mental state examination (MMSE) was used to evaluate general cognition (29); the verbal fluency test (VFT) was used to evaluate executive function. Digit span-forward (DS-F) and -backward (DS-B) tests were used to estimate working memory. The digit symbol substitution test (DSST) was used to evaluate the information-processing speed (30). All neuropsychological parameters were estimated before the image scan.

### 2.3. Imaging data

Imaging was performed at the Third Affiliated Hospital of Anhui Medical University using a Siemens Verio 3.0-Tesla scanner (Siemens, Erlangen, Germany). MRI data acquisition was performed between 17:00 and 19:00 for all participants. Structural images were acquired using a high-resolution spoiled gradient-recalled echo sequence with the following parameters: repetition time/echo time (TR/TE) = 1,900/2.48 ms; flip angle (FA) = 9°, acquisition matrix = 256 × 256, field-of-view = 240 × 240 mm, thickness = 1.0 mm; gap = 0 mm, number of slices = 176, and number of excitations = 1.0. The rs-fMRI datasets were obtained using an 8-min gradient-recalled echo-planar imaging pulse sequence with the following parameters: TR/TE, 2,000/35 ms; FA, 90°; acquisition matrix, 64 × 64; thickness = 3.5 mm; number of slices = 36, number of time points, 240. During scanning, all participants were instructed to relax and keep their eyes closed for the whole sequence, and stabilizers were used to immobilize their heads. Wakefulness was assessed following the scanning, and all participants were awake during the study.

The rs-fMRI data were preprocessed using SPM12^1^ and DPABI 4.3 (Data Processing & Analysis of Brain Imaging^2^) implemented in MATLAB 8.0 (The MathWorks, Inc., Natick, MA, USA) (31). We removed the first five initial volumes to account for the magnetization equilibrium and adaptation to the experimental environment. The remaining 235 images were then slice-time corrected, reoriented, realigned, and co-registered to the T1-weighted structural images, which were segmented using DARTEL (32). Images from all participants were normalized into standard stereotactic Montreal Neurological Institute space and smoothed using a Gaussian kernel (full width at half-maximum = 6 mm). The voxel time series was further detrended and temporally filtered (0.01–0.1 Hz). We normalized the variance of each time series to control for fluctuations in the signal intensity. Noise associated with white matter/cerebrospinal fluid signals and 24 head motion-related covariates were regressed. The mean framewise displacement was not significant between the two groups (*p* > 0.05). The global signal was not regressed, given the controversy regarding its application to rs-fMRI data (33, 34).

### 2.4. Habenula-based FC network construction

The habenula shows structural and functional asymmetry (11). Therefore, the bilateral habenular nucleus was selected according to the myelin content-based human habenula segmentation and separately centered for the left habenula (−2.7, −24.3, 2.2) and right habenula (4.0, −23.6, 2.2) (35). Seed-based voxel-wise FC analysis was employed to construct the habenular FC network using the DPABI 4.3 toolbox. First, the average time course in each habenula region was extracted and defined as the seed time series. Second, the correlation between the seed region and all voxels in the other brain regions was calculated using Pearson’s correlation analyses. Third, Fisher’s *Z*-transformation was applied to improve the correlation coefficients so that they approached normal distribution [*Z* = 0.5 ln (1 + CC)/(1 − CC)]. Finally, each participant’s habenular FC network was obtained for the next analyses.

### 2.5. Statistical analyses

First, two-sample *t*-tests and chi-square tests were conducted for the demographic and neuropsychological test comparisons between the CID and GSC groups (SPSS 24.0; SPSS Inc., Chicago, IL, USA). The relationships between clinical features and neuropsychological tests were calculated using Pearson’s correlation analyses, and the effects of age, sex, and education were controlled. The significance threshold was set at a *p* < 0.05, and the multiple comparison correction was conducted using False Discovery Rate (FDR) approach.

Second, a voxel-wise, one-sample *t*-test was used to obtain the bilateral habenular FC network pattern in each group, and the voxel-level significance threshold was set at an uncorrected value of *p* <0.001.

Third, two independent t-tests were used to obtain group differences in habenular FC networks, controlling for the effects of sex and age. We used permutation testing with threshold-free cluster enhancement (TFCE) to threshold our results at *p* < 0.05. TFCE was conducted in the permutation analysis of linear models package in DPABI with 5,000 permutations and a cluster-forming threshold of *z* > 2.3.

Finally, to further explore the clinical and neuropsychological significance of abnormal habenular FC in patients with CID, a partial correlation analysis was used to investigate the association between altered habenular FC and clinical and neuropsychological features (including PSQI, ISS, ESS, HAS, SAS, SDS, MMSE, VFT, DS-F, DS-B, and DSST scores), controlling for the effects of age, sex, and disease duration. Significance was set at a *p* < 0.05, and the multiple comparison correction was conducted using the FDR approach.

## 3. Results

### 3.1. Demographic and neuropsychological information

Table 1 shows the demographic and neuropsychological information of the two groups. The basic demographic data were matched between the two groups in terms of age, sex, and education (*p* > 0.05). The mean and standard deviation of the duration of insomnia were 7.22 and 4.07, respectively. The PSQI, ISI, SDS, and SAS scores were higher in patients with CID than in those in the GSC group (all *p* < 0.01, p-FDR of PSQI, ISI, SAS < 0.001, p-FDR of SDS = 0.028). There was a trend-wise significant difference in hyperarousal state (HAS) between CID and GSC (*t* = 1.85, *p* = 0.07). According to previous findings, HAS >29 indicates a hyperarousal state in insomnia patients (36); as such, the ratio of patients with hyperarousal was higher in the CID group than in the GSC group (CID: 26/6, GSC: 8/12, Chi-Square is 9.25, *p* = 0.003). In addition, no significant differences were found in subjective daytime sleepiness (ESS) or cognitive performance (including MMSE, VFT, DS, or DSST) (all *p* > 0.05).

**Table 1:** Demographic and clinical traits for all participants.

Characteristic	CID (*n* = 32)	GSC (n = 20)	*T/X^2^*	Value of *p*
Age	41.12 ± 13.72	38.45 ± 16.15	0.64	0.53
Sex (Male/Female)	22/10	12/8	0.42	0.56
Education(years)	12.84 ± 4.07	12.85 ± 4.43	0.001	0.99
Duration (years)	7.22 ± 6.53	-	-	-
PSQI	12.96 ± 3.51	3.95 ± 1.61	10.76	<0.001
ISI	16.31 ± 6.11	4.00 ± 3.45	8.20	<0.001
ESS	6.62 ± 5.27	7.95 ± 4.87	0.91	0.37
HAS	40.06 ± 13.91	33.60 ± 9.76	1.85	0.07
SDS	48.20 ± 13.15	38.31 ± 11.76	2.75	0.008
SAS	47.07 ± 11.67	35.00 ± 6.32	4.24	<0.001
MMSE	28.12 ± 1.84	28.80 ± 1.79	1.29	0.20
VFT	17.37 ± 6.62	15.15 ± 5.72	1.24	0.22
DS-F	8.75 ± 1.43	9.25 ± 1.21	1.29	0.20
DS-B	5.28 ± 1.87	5.20 ± 1.76	0.16	0.88
DSST	44.75 ± 19.10	47.05 ± 22.59	0.39	0.69
Mean FD	0.32 ± 0.65	0.20 ± 0.17	0.82	0.42

CID, chronic insomnia disorder; GSC, good sleep control; PSQI, Pittsburgh Sleep Quality Index; ISI, insomnia severity index scale; ESS, Epworth sleepiness scale; HAS, hyperarousal scale; SDS, Zung Self-Depression Scale; SAS, Zung self-anxiety scale; MMSE, Mini-Mental State Examination; VFT, verbal fluency test; DS-F, digit span-forward; DS-B, digit span-backward; DSST, digit symbol substitution test; FD, framewise displacement.

**Figure 1:**
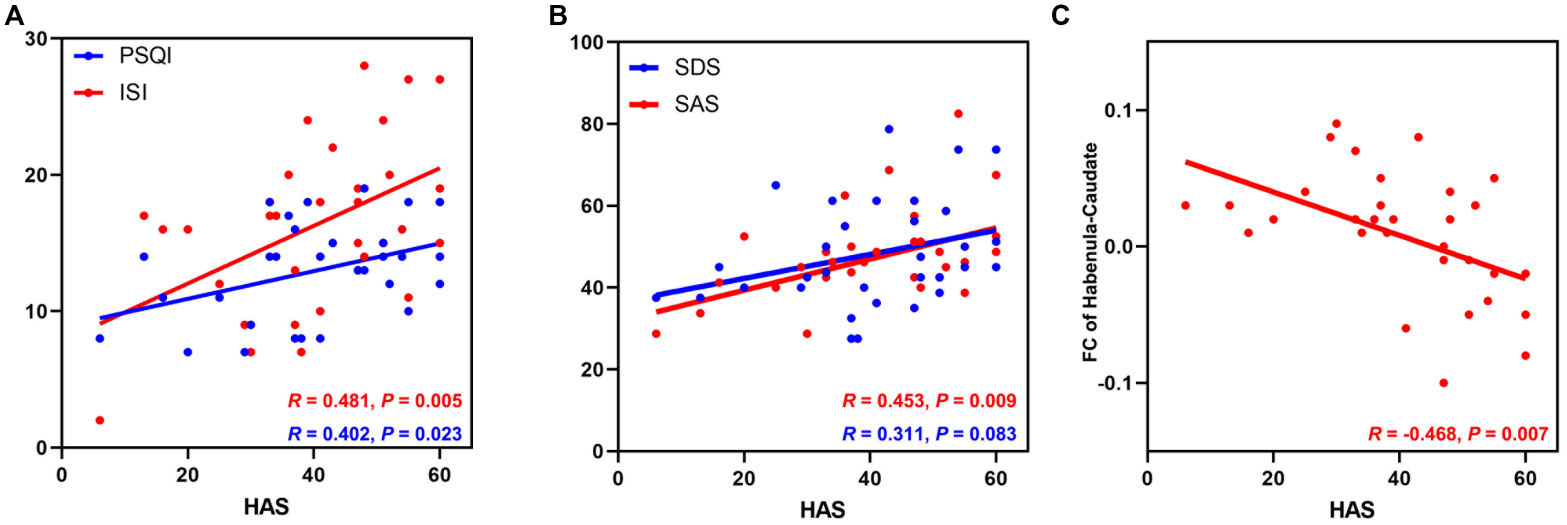
The relationship between HAS scores and clinical features and altered habenular FC in patients with CID. **(A)** The HAS score was positively associated with the severity of insomnia; **(B)** The HAS score was positively associated with anxiety symptoms but not depressive symptoms in patients with CID. **(C)** The HAS score was negatively associated with the abnormal FC between the left habenula and left caudate nucleus in the CID group. CID, chronic insomnia disorder; HAS, hyperarousal scale; PSQI, Pittsburgh Sleep Quality Index; ISI, Insomnia Severity Index; SDS, Zung self-depression scale; SAS, Zung self-anxiety scale; CAU, caudate nucleus; FC, functional connectivity.

The correlations between the clinical features and neuropsychological tests in the CID group are presented in Table 2. Specifically, the severity of insomnia (PSQI and ISI scores) was significantly positively associated with HAS, anxiety, and depression symptoms (SAS and SDS), but not with cognitive performance in the CID group. In addition, the HAS was positively associated with anxiety symptoms (*p* = 0.009) but not with depression symptoms in the CID group (*p* = 0.08) After applying multiple comparison corrections, none of the correlations were statistically significant (uncorrected statistical values shown in Figures 1A,B).

**Table 2:** Correlations between clinical features and neuropsychological tests in the chronic insomnia disorder group.

		Duration	PSQI	ISI	ESS	SDS	SAS	HAS	MMSE	VFT	DS-F	DS-B	DSST
Duration	*r*	1	−0.109	−0.122	−0.068	−0.084	−0.028	0.193	−0.31	−0.25	−0.29	−0.294	−0.206
	*p*		0.554	0.506	0.71	0.649	0.877	0.289	0.084	0.168	0.107	0.103	0.258
PSQI	*r*	−0.109	1	0.767^**^	−0.298	0.400^*^	0.407^*^	0.402^*^	0.205	−0.185	−0.008	0.065	−0.139
	*p*	0.554		0	0.097	0.023	0.021	0.023	0.261	0.31	0.965	0.723	0.45
ISI	*r*	−0.122	0.767^**^	1	0.01	0.381^*^	0.410^*^	0.481^**^	0.062	−0.157	−0.009	0.043	−0.164
	*p*	0.506	0		0.958	0.031	0.02	0.005	0.735	0.39	0.96	0.816	0.371
ESS	*r*	−0.068	−0.298	0.01	1	−0.091	−0.034	−0.001	−0.018	−0.025	0.179	−0.035	−0.143
	*p*	0.71	0.097	0.958		0.621	0.853	0.997	0.921	0.89	0.327	0.85	0.435
SDS	*r*	−0.084	0.0400^*^	0.381^*^	−0.091	1	0.644^**^	0.311	0.242	−0.277	0.069	0.146	0.118
	*p*	0.649	0.023	0.031	0.621		0	0.083	0.182	0.125	0.706	0.426	0.52
SAS	*r*	−0.028	0.407^*^	0.410^*^	−0.034	0.644^**^	1	0.453^**^	0.225	−0.188	0.166	−0.037	−0.064
	*p*	0.877	0.021	0.02	0.853	0		0.009	0.216	0.303	0.363	0.842	0.729
HAS	*r*	0.193	0.402^*^	0.481^**^	−0.001	0.311	0.453^**^	1	0.043	−0.037	−0.052	−0.227	−0.162
	*p*	0.289	0.023	0.005	0.997	0.083	0.009		0.815	0.84	0.776	0.213	0.375
MMSE	*r*	−0.31	0.205	0.062	−0.018	0.242	0.225	0.043	1	0.242	0.621^**^	0.261	0.579^**^
	*p*	0.084	0.261	0.735	0.921	0.182	0.216	0.815		0.183	0	0.15	0.001
VFT	*r*	−0.25	−0.185	−0.157	−0.025	−0.277	−0.188	−0.037	0.242	1	0.231	0.491^**^	0.429^*^
	*p*	0.168	0.31	0.39	0.89	0.125	0.303	0.84	0.183		0.204	0.004	0.014
DS-F	*r*	−0.29	−0.008	−0.009	0.179	0.069	0.166	−0.052	0.621^**^	0.231	1	0.519^**^	0.634^**^
	*p*	0.107	0.965	0.96	0.327	0.706	0.363	0.776	0	0.204		0.002	0
DS-B	*r*	−0.294	0.065	0.043	−0.035	0.146	−0.037	−0.227	0.261	0.491^**^	0.519^**^	1	0.537^**^
	*p*	0.103	0.723	0.816	0.85	0.426	0.842	0.213	0.15	0.004	0.002		0.002
DSST	*r*	−0.206	−0.139	−0.164	−0.143	0.118	−0.064	−0.162	0.579^**^	0.429^*^	0.634^**^	0.537^**^	1
	*p*	0.258	0.45	0.371	0.435	0.52	0.729	0.375	0.001	0.014	0	0.002	

*p < 0.05; **p < 0.01. PSQI, Pittsburgh sleep quality index; ISI, insomnia severity index scale; ESS, Epworth sleepiness scale; HAS, hyperarousal scale; SDS, Zung self-depression scale; SAS, Zung self-anxiety scale; MMSE, mini-mental state examination; VFT, verbal fluency test; DS-F, digitspan-forward; DS-B, digit span-backward; DSST, digit symbol substitution test.

### 3.2. Bilateral habenular FC network patterns in both groups

**Figure 2:**
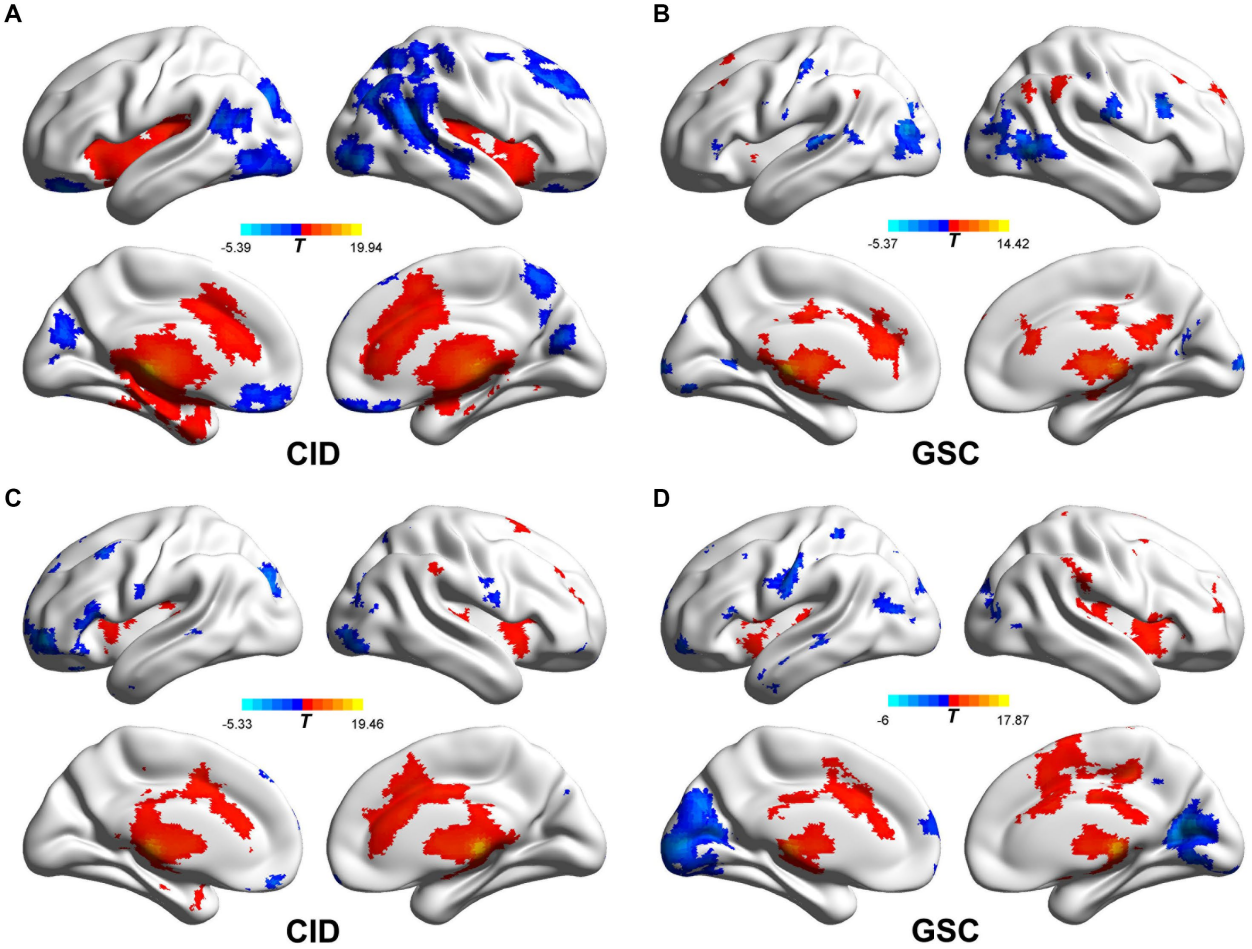
Functional connectivity patterns of the bilateral habenula in CID and GSC groups. **(A,B)** The left habenular functional connectivity pattern of CID and GSC groups, respectively. **(C,D)** The right habenular functional connectivity pattern of CID and GSC groups, respectively. CID, chronic insomnia disorder; GSC, good sleep control.

The intrinsic FC network patterns of the habenula are shown in Figure 2. The habenular FC network was similar between the two groups, and the habenular FC pattern was similar to that in a previous report (21). The habenula-positive connected brain regions were located in the subcortical region (caudate, pallidum, nucleus accumbens, putamen, and thalamus), sensorimotor cortex, insula, dorsal anterior cingulate cortex, posterior cingulate cortex, and medial temporal gyrus. The habenula-negative connected brain regions were found in the orbitofrontal cortex, dorsomedial prefrontal cortex, precuneus, and occipital cortex.

### 3.3. Altered habenular FC network in the CID group

**Figure 3:**
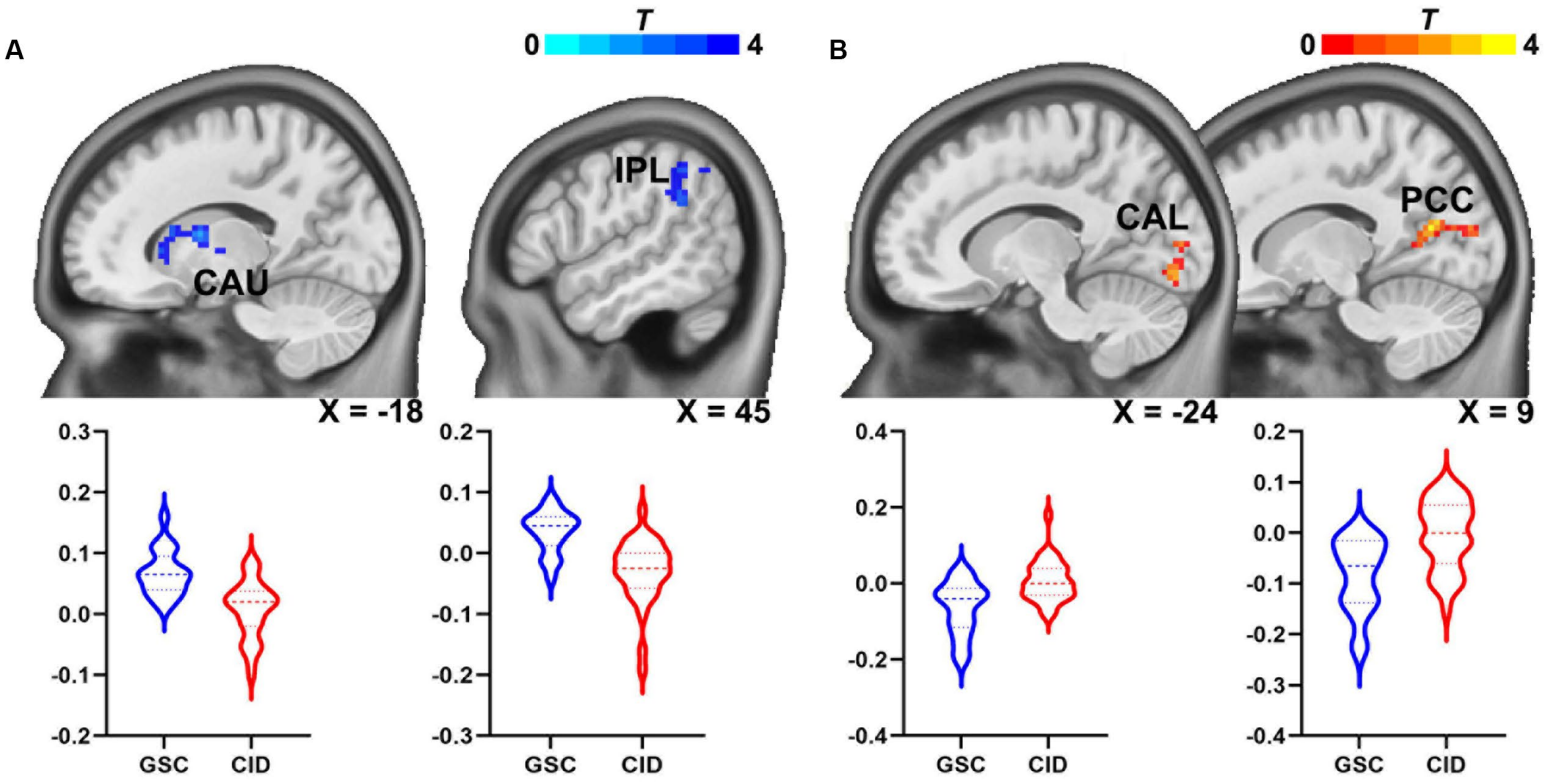
Abnormal habenular functional network in patients with CID. **(A)** Connectivity of the left habenular functional network in the left caudate nucleus and right IPL was reduced in patients with CID. **(B)** Connectivity in the right habenular functional network in patients with CID was increased in the left calcarine and right calcarine/PCC. CAU, caudate nucleus; CID, chronic insomnia disorder; IPL, inferior parietal lobule; CAL, calcarine cortex; PCC, posterior cingulate cortex.

The group differences in the bilateral habenular functional networks are illustrated in Table 3 and Figure 3. In the left habenular FC network, FC in the left caudate and right inferior parietal lobule was decreased in the CID group relative to the GSC group. In the right habenular FC network, FC in the left calcarine and right calcarine/posterior cingulate cortex (PCC) were increased in the CID group relative to the GSC group. No significant group differences in the bilateral habenular functional networks were found between CID patients with or without hyperarousal state after multiple correlations; we presented the group differences map without multiple comparations (*p* < 0.05, uncorrected, Supplementary Figure S1).

**Table 3:** Group differences in the bilateral habenular FC network.

Brain region	BA	Voxel size	MNI coordinates (RAI)			Peak *T* score
			*x*	*y*	*z*	
Left habenular FC network						
Left caudate nucleus	-	111	−18	21	0	−4.18
Right IPL	39/40	148	45	−63	39	−3.96
Right habenular FC network						
Left calcarine cortex	17	94	−24	−84	−6	3.52
Right calcarine cortex/PCC	18	99	9	−66	15	4.01

FC, functional connectivity; BA, Brodmann area; IPL, inferior parietal lobule; PCC, posterior cingulate cortex.

### 3.4. Clinical significance of the altered habenular FC network

As shown in Figure 1C, the FC between the left habenula and left caudate nucleus was negatively correlated with the HAS (HAS scores, *R* = −0.468, *p* = 0.007) in patients with CID after controlling for the effects of sex, age, and education; after multiple comparations, this correction was not significant (p-FDR = 0.147). No other significant association was found between altered habenular FC and clinical features or cognitive performance.

## 4. Discussion

We investigated whether habenular function was altered in patients with CID based on the seed-based FC method. Compared to good sleepers, the results showed that the left habenular FC decreased in the left caudate nucleus and right IPL, while the right habenular FC increased in the bilateral calcarine and PCC in patients with CID. In addition, the decreased habenular FC in the left caudate nucleus was associated with an increased HAS in patients with CID. These findings indicate that habenular function is abnormal in patients with CID and that the abnormal habenular functional network may be involved in the hyperarousal neurobiological mechanism underlying chronic insomnia. Thus, our findings provide evidence for the brain mechanism of the hyperarousal process model of chronic insomnia.

The caudate nucleus is the core region of the striatum and is involved in goal-directed action and positive reward processing (37). Additionally, the lateral habenula has been demonstrated as a key brain region for reward regulation (38). The lateral habenula has been known to modulate DA release and process nociceptive signals in the mesolimbic DA system (39). Moreover, recent electrophysiological studies provide evidence that prefrontal and non-prefrontal cortical regions can indirectly convey information to the lateral habenula *via* a cortico-striosome-pallido-habenular circuit in macaque monkeys (40). The dopamine neurons in the habenula encode negative reward-related signals and reward prediction errors (41).

The dysfunctional connectivity between these two regions extends our understanding of the abnormal reward processing theory in chronic insomnia (42) and is consistent with our previous findings. First, the nucleus accumbens-based reward network is altered in CID (7), and second, the inferior parietal lobule is an important node in the posterior of the default mode network (DMN) (43), and third bottom-up perception plays an important role in integrating somatosensory, visual, and auditory information (44). Thus, the decreased FC between the habenula and IPL might indicate abnormal sensory integration in patients with CID.

Furthermore, increased habenular FC was found in the bilateral calcarine cortex and PCC in CID participants. The PCC is also the core code in the posterior of the DMN, implicated in both arousal state and self-referential processes (45). Increased FC between the habenula and PCC has also been reported in patients with mood disorders, with and without suicide-related behaviors (46), and abnormal and unbalanced DMN functions have been reported in a previous study on sleep disorders, including chronic insomnia disorder (8). Our results indicate that connectivity between the posterior DMN and the habenula was also imbalanced in patients with CID. The calcarine cortex is also known as the primary visual cortex or V1, which is the first site for visual stimulus processing (47). The increased FC between the habenula and calcarine cortex is consistent with the hyperarousal process model in the visual cortex in the neuropathology of CID. Taken together, our findings indicate an abnormal habenular function in patients with CID and contribute evidence that may help elucidate the underlying neurobiological mechanism of insomnia and the HAS in CID.

The hyperarousal scale is a subjective evaluation to measure an individual arousal state during the daytime and is used in conjunction with the objective arousal state measured using EEG (48), whereas, the activity of the autonomic nervous system (heart rate variability and heart rate) can be used as an indicator to evaluate the objective state of hyperarousal levels, and these pieces of evidence imply an overactivity of these systems in insomnia, supporting the assumption that hyperarousal states contribute to insomnia in these patients (49).

Previous studies have shown that HAS scores reflect higher alertness in insomnia patients than in good sleepers (48). In our study, the HAS score did not differ significantly between the CID and GSC groups, but there was a tendency for CID participants to have higher HAS scores than GSC participants. This result may be attributed to the small sample in this study. The HAS score was positively associated with the severity of insomnia and anxiety symptoms but not with depression or cognitive performance in the CID group (Figure 1). These results are similar to those of a previous study on insomnia and anxiety (50). Epidemiological studies show that sleep disturbances, particularly insomnia, affect 50% of patients with anxiety (51), which is consistent with previous research suggesting that anxiety is associated with a high arousal state before bed (52). In addition, we found that decreased FC between the left habenula and caudate nucleus was associated with the HAS in the CID group. Previous evidence indicates the absence of the negative effects of melatonin ingestion on vigilance and arousal (53). In addition, mice subjected to sleep deprivation had significantly decreased plasma melatonin content (54). Although the habenula regulates melatonin receptors, it affects melatonin secretion in the pineal gland (55). However, whether the habenular nucleus regulates melatonin levels to maintain elevated arousal is unclear.

As mentioned above, both the habenula and caudate nucleus are involved in the reward-processing network. Recent evidence has identified that the hypothalamic neurons are involved in both reward and arousal processing regulated by the neuropeptide transmitter orexin (56), while the habenular norepinephrine system contributes to the arousal and anxiety state (56). Therefore, we speculate that the HAS in insomnia might be associated with habenula-related abnormal neurotransmitters; however, further studies are required to verify our hypothesis. Taken together, these findings may indicate that the habenula is a potential target for HAS modulation in patients with CID.

Our findings reflected the habenular functional asymmetry and asymmetry dysfunction in patients with CID (11). Patients with CID showed decreased FC in the left habenular functional network, while FC in the right habenular functional network increased. Interestingly, the decreased FC between the left habenula and left caudate nucleus was significantly associated with the HAS, whereas the right habenula was not involved in patients with CID. Recently, Ambrosi et al. found left asymmetric habenular function in patients with suicidal mood disorder (46). Our results suggest that habenular left-hemispheric asymmetry may be important for the hyperarousal model of insomnia neurobiology. These findings imply that hemispheric asymmetry should be considered in habenula function research of psychiatric disorders.

### 4.1. Limitations

Our study had some limitations. First, the preliminary study had a small sample and a cross-sectional design, which resulted in non-significant correlations between clinical features and neuropsychological tests, as well as altered habenula FC, after multiple comparison corrections. Thus, future studies with larger samples are needed to confirm our findings. Second, we used only self-evaluation scales for arousal state in the study; a neurotic electrophysiology-based arousal evaluation may provide a more comprehensive understanding of the brain mechanism underlying the HAS in CID. Third, there are two subnuclei in the human habenula, the medial and lateral habenula, which have distinct neuroanatomical and FC with other brain regions (11). Future studies using higher resolution functional MRI may be beneficial in exploring the subnuclei habenular functional alteration in CID. Fourth, patients with CID were not tested with polysomnography, and subtypes of chronic insomnia, such as difficulty falling and maintaining sleep, early morning awakening, and non-restorative sleep were not grouped. Therefore, whether there are differences in brain function changes among different subtypes of chronic insomnia requires further research.

## 5. Conclusion

Our study provides evidence of a dysfunctional habenular network in patients with CID. In addition, altered FC between the habenula and caudate nucleus may be associated with the HAS in the CID group. Our findings can be used as reference in understanding the neuropathological mechanisms underlying the hyperarousal model in chronic insomnia.

## Data availability statement

The raw data supporting the conclusions of this article will be made available by the authors, without undue reservation.

## Ethics statement

The studies involving human participants were reviewed and approved by the ethical committee of the Institutional Review Board of the Third Affiliated Hospital of Anhui Medical University. The patients/participants provided their written informed consent to participate in this study.

## Author contributions

CX, RX, BZ, and LG were responsible for study design, statistical analyses, and manuscript preparation. CX and LG analyzed the data and wrote a manuscript. FC and XL were responsible for the collection of participants’ information. ZW and SW participated in the editing and submission of the manuscript. All authors had full access to all the data in the study and take responsibility for the integrity of the data and the accuracy of the data analyses, contributed to and have approved the final manuscript.

## Funding

This study was funded by the Basic and clinical cooperative research Program of the Anhui Medical University-incubation project for the Third Affiliated Hospital (2022sfy001), the Applied Medicine Research Project of Hefei Municipal Health Commission (Hwk2019zd003 and Hwk2022zd002), the Scientific Research Fund project of Anhui Medical University (2021xkj222), Clinical Medical Research Transformation Project of Anhui Provincial Science and Technology Department (202204295107020024), the Sichuan Provincial Science and Technology Department project (2020YJ0197), the National Natural Science Foundation of China (82001803), and the Chengdu Science and Technology project (2021-YF05-00247-SN).

## Conflict of interest

The authors declare that the research was conducted in the absence of any commercial or financial relationships that could be construed as a potential conflict of interest.

The reviewer JZ declared a shared affiliation with the authors to the handling editor at the time of review.

## Publisher’s note

All claims expressed in this article are solely those of the authors and do not necessarily represent those of their affiliated organizations, or those of the publisher, the editors and the reviewers. Any product that may be evaluated in this article, or claim that may be made by its manufacturer, is not guaranteed or endorsed by the publisher.

## Footnotes

^1^http://www.fil.ion.ucl.ac.uk/spm

^2^http://rfmri.org/dpabi

## References

[1] Buysse DJ. Insomnia. JAMA. (2013) 309:706–16. doi: 10.1001/jama.2013.193, PMID: 23423416 PMC3632369

[2] Blake MJ Trinder JA Allen NB. Mechanisms underlying the association between insomnia, anxiety, and depression in adolescence: implications for behavioral sleep interventions. Clin Psychol Rev. (2018) 63:25–40. doi: 10.1016/j.cpr.2018.05.006, PMID: 29879564

[3] Nofzinger EA Buysse DJ Germain A Price JC Miewald JM Kupfer DJ. Functional neuroimaging evidence for hyperarousal in insomnia. Am J Psychiatry. (2004) 161:2126–8. doi: 10.1176/appi.ajp.161.11.2126, PMID: 15514418

[4] Spiegelhalder K Riemann D. Hyperarousal and insomnia. Sleep Med Clin. (2013) 8:299–307. doi: 10.1016/j.jsmc.2013.04.008, PMID: 37421322

[5] Spiegelhalder K Regen W Baglioni C Riemann D Winkelman JW. Neuroimaging studies in insomnia. Curr Psychiatry Rep. (2013) 15:405. doi: 10.1007/s11920-013-0405-0 24057158

[6] Gong L Liao T Liu D Luo Q Xu R Huang Q. Amygdala changes in chronic insomnia and their association with sleep and anxiety symptoms: insight from shape analysis. Neural Plast. (2019) 2019:1–8. doi: 10.1155/2019/8549237, PMID: 31885536 PMC6914992

[7] Gong L Yu S Xu R Liu D Dai X Wang Z. The abnormal reward network associated with insomnia severity and depression in chronic insomnia disorder. Brain Imaging Behav. (2021) 15:1033–42. doi: 10.1007/s11682-020-00310-w, PMID: 32710331

[8] Yu S Guo B Shen Z Wang Z Kui Y Hu Y. The imbalanced anterior and posterior default mode network in the primary insomnia. J Psychiatr Res. (2018) 103:97–103. doi: 10.1016/j.jpsychires.2018.05.013, PMID: 29804003

[9] Gong L He K Cheng F Deng Z Cheng K Zhang X. The role of ascending arousal network in patients with chronic insomnia disorder. Hum Brain Mapp. (2023) 44:484–95. doi: 10.1002/hbm.26072, PMID: 36111884 PMC9842899

[10] Namboodiri VM Rodriguez-Romaguera J Stuber GD. The habenula. Curr Biol. (2016) 26:R873–7. doi: 10.1016/j.cub.2016.08.051, PMID: 27728786

[11] Aizawa H Amo R Okamoto H. Phylogeny and ontogeny of the habenular structure. Front Neurosci. (2011) 5:138. doi: 10.3389/fnins.2011.00138, PMID: 22203792 PMC3244072

[12] Evely KM Hudson RL Dubocovich ML Haj-Dahmane S. Melatonin receptor activation increases glutamatergic synaptic transmission in the rat medial lateral habenula. Synapse. (2016) 70:181–6. doi: 10.1002/syn.21892 26799638

[13] Shabel Steven J Proulx Christophe D Piriz J Malinow R. GABA/glutamate co-release controls habenula output and is modified by antidepressant treatment. Science. (2014) 345:1494–8. doi: 10.1126/science.1250469, PMID: 25237099 PMC4305433

[14] Hu H Cui Y Yang Y. Circuits and functions of the lateral habenula in health and in disease. Nat Rev Neurosci. (2020) 21:277–95. doi: 10.1038/s41583-020-0292-4, PMID: 32269316

[15] Speranza L di Porzio U Viggiano D de Donato A Volpicelli F. Dopamine: the neuromodulator of long-term synaptic plasticity, reward and movement control. Cells. (2021) 10:735. doi: 10.3390/cells10040735, PMID: 33810328 PMC8066851

[16] Lazaridis I Tzortzi O Weglage M Märtin A Xuan Y Parent M. A hypothalamus-habenula circuit controls aversion. Mol Psychiatry. (2019) 24:1351–68. doi: 10.1038/s41380-019-0369-5, PMID: 30755721 PMC6756229

[17] Pribiag H Shin S Wang EH Sun F Datta P Okamoto A. Ventral pallidum DRD3 potentiates a pallido-habenular circuit driving accumbal dopamine release and cocaine seeking. Neuron. (2021) 109:2165–2182.e10. doi: 10.1016/j.neuron.2021.05.002, PMID: 34048697 PMC9013317

[18] Lee YA Kim YJ Lee JS Lee S Goto Y. Imbalance between dopamine and serotonin caused by neonatal habenula lesion. Behav Brain Res. (2021) 409:113316. doi: 10.1016/j.bbr.2021.113316, PMID: 33901435

[19] Lawson RP Nord CL Seymour B Thomas DL Dayan P Pilling S. Disrupted habenula function in major depression. Mol Psychiatry. (2017) 22:202–8. doi: 10.1038/mp.2016.81, PMID: 27240528 PMC5285459

[20] Aizawa H Cui W Tanaka K Okamoto H. Hyperactivation of the habenula as a link between depression and sleep disturbance. Front Hum Neurosci. (2013) 7:826. doi: 10.3389/fnhum.2013.00826, PMID: 24339810 PMC3857532

[21] Ely BA Xu J Goodman WK Lapidus KA Gabbay V Stern ER. Resting-state functional connectivity of the human habenula in healthy individuals: associations with subclinical depression. Hum Brain Mapp. (2016) 37:2369–84. doi: 10.1002/hbm.23179, PMID: 26991474 PMC4905808

[22] Mao CP Chen FR Huo JH Zhang L Zhang GR Zhang B. Altered resting-state functional connectivity and effective connectivity of the habenula in irritable bowel syndrome: a cross-sectional and machine learning study. Hum Brain Mapp. (2020) 41:3655–66. doi: 10.1002/hbm.25038, PMID: 32488929 PMC7416021

[23] Zhu Y Qi S Zhang B He D Teng Y Hu J. Connectome-based biomarkers predict subclinical depression and identify abnormal brain connections with the lateral habenula and thalamus. Front Psych. (2019) 10:371. doi: 10.3389/fpsyt.2019.00371, PMID: 31244688 PMC6581735

[24] Sateia MJ. International classification of sleep disorders-third edition: highlights and modifications. Chest. (2014) 146:1387–94. doi: 10.1378/chest.14-0970, PMID: 25367475

[25] Backhaus J Junghanns K Broocks A Riemann D Hohagen F. Test-retest reliability and validity of the Pittsburgh sleep quality index in primary insomnia. J Psychosom Res. (2002) 53:737–40. doi: 10.1016/S0022-3999(02)00330-6, PMID: 12217446

[26] Morin CM Belleville G Bélanger L Ivers H. The insomnia severity index: psychometric indicators to detect insomnia cases and evaluate treatment response. Sleep. (2011) 34:601–8. doi: 10.1093/sleep/34.5.601, PMID: 21532953 PMC3079939

[27] Hammad MA Barsky AJ Regestein QR. Correlation between somatic sensation inventory scores and hyperarousal scale scores. Psychosomatics. (2001) 42:29–34. doi: 10.1176/appi.psy.42.1.29, PMID: 11161118

[28] Johns MW. A new method for measuring daytime sleepiness: the Epworth sleepiness scale. Sleep. (1991) 14:540–5. doi: 10.1093/sleep/14.6.540, PMID: 1798888

[29] Kurlowicz L Wallace M. The mini-mental state examination (MMSE). Thorofare, NJ: SLACK Incorporated (1999). 10.3928/0098-9134-19990501-08 10578759

[30] McLeod DR Griffiths RR Bigelow GE Yingling J. An automated version of the digit symbol substitution test (DSST). Behav Res Methods Instrum. (1982) 14:463–6. doi: 10.3758/BF03203313, PMID: 3172783

[31] Yan CG Wang XD Zuo XN Zang YF. DPABI: Data Processing & Analysis for (resting-state) brain imaging. Neuroinformatics. (2016) 14:339–51. doi: 10.1007/s12021-016-9299-4, PMID: 27075850

[32] Ashburner J. A fast diffeomorphic image registration algorithm. NeuroImage. (2007) 38:95–113. doi: 10.1016/j.neuroimage.2007.07.007, PMID: 17761438

[33] Chai XJ Castañón AN Öngür D Whitfield-Gabrieli S. Anticorrelations in resting state networks without global signal regression. NeuroImage. (2012) 59:1420–8. doi: 10.1016/j.neuroimage.2011.08.048, PMID: 21889994 PMC3230748

[34] Murphy K Birn RM Handwerker DA Jones TB Bandettini PA. The impact of global signal regression on resting state correlations: are anti-correlated networks introduced? NeuroImage. (2009) 44:893–905. doi: 10.1016/j.neuroimage.2008.09.036, PMID: 18976716 PMC2750906

[35] Kim JW Naidich TP Joseph J Nair D Glasser MF O'halloran R. Reproducibility of myelin content-based human habenula segmentation at 3 tesla. Hum Brain Mapp. (2018) 39:3058–71. doi: 10.1002/hbm.24060, PMID: 29582505 PMC6033622

[36] Khassawneh BY Bathgate CJ Tsai SC Edinger JD. Neurocognitive performance in insomnia disorder: the impact of hyperarousal and short sleep duration. J Sleep Res. (2018) 27:e12747. doi: 10.1111/jsr.12747, PMID: 30069961

[37] Schultz W. Reward functions of the basal ganglia. J Neural Transm (Vienna). (2016) 123:679–93. doi: 10.1007/s00702-016-1510-0, PMID: 26838982 PMC5495848

[38] Mondoloni S Mameli M Congiu M. Reward and aversion encoding in the lateral habenula for innate and learned behaviours. Transl Psychiatry. (2022) 12:3. doi: 10.1038/s41398-021-01774-0 35013094 PMC8748902

[39] Lee SM Jang HB Fan Y Lee BH Kim SC Bills KB. Nociceptive stimuli activate the hypothalamus-habenula circuit to inhibit the mesolimbic reward system and cocaine-seeking behaviors. J Neurosci. (2022) 42:9180–92. doi: 10.1523/JNEUROSCI.0577-22.2022, PMID: 36280259 PMC9761669

[40] Hong S Amemori S Chung E Gibson DJ Amemori KI Graybiel AM. Predominant striatal input to the lateral habenula in macaques comes from striosomes. Curr Biol. (2019) 29:51–61.e5. doi: 10.1016/j.cub.2018.11.008, PMID: 30554903 PMC6561345

[41] Matsumoto M Hikosaka O. Lateral habenula as a source of negative reward signals in dopamine neurons. Nature. (2007) 447:1111–5. doi: 10.1038/nature05860, PMID: 17522629

[42] Motomura Y Katsunuma R Ayabe N Oba K Terasawa Y Kitamura S. Decreased activity in the reward network of chronic insomnia patients. Sci Rep. (2021) 11:3600. doi: 10.1038/s41598-020-79989-2, PMID: 33574355 PMC7878866

[43] Buckner RL Andrews-Hanna JR Schacter DL. The brain's default network: anatomy, function, and relevance to disease. Ann N Y Acad Sci. (2008) 1124:1–38. doi: 10.1196/annals.1440.011 18400922

[44] Caspers S Schleicher A Bacha-Trams M Palomero-Gallagher N Amunts K Zilles K. Organization of the Human Inferior Parietal Lobule Based on receptor architectonics. Cereb Cortex. (2013) 23:615–28. doi: 10.1093/cercor/bhs048, PMID: 22375016 PMC3563340

[45] Raichle ME. The brain's default mode network. Annu Rev Neurosci. (2015) 38:433–47. doi: 10.1146/annurev-neuro-071013-014030, PMID: 25938726

[46] Ambrosi E Arciniegas DB Curtis KN Patriquin MA Spalletta G Sani G. Resting-state functional connectivity of the habenula in mood disorder patients with and without suicide-related Behaviors. J Neuropsychiatry Clin Neurosci. (2019) 31:49–56. doi: 10.1176/appi.neuropsych.17120351, PMID: 30282513 PMC6697145

[47] Meadows M-E. Calcarine Cortex In: Kreutzer JS DeLuca J Caplan B, editors. Encyclopedia of clinical neuropsychology. New York, New York, NY: Springer (2011)

[48] Pavlova M Berg O Gleason R Walker F Roberts S Regestein Q. Self-reported hyperarousal traits among insomnia patients. J Psychosom Res. (2001) 51:435–41. doi: 10.1016/S0022-3999(01)00189-1, PMID: 11516766

[49] Riemann D Nissen C Palagini L Otte A Perlis ML Spiegelhalder K. The neurobiology, investigation, and treatment of chronic insomnia. Lancet Neurol. (2015) 14:547–58. doi: 10.1016/S1474-4422(15)00021-6, PMID: 25895933

[50] Tang NK Harvey AG. Effects of cognitive arousal and physiological arousal on sleep perception. Sleep. (2004) 27:69–78. doi: 10.1093/sleep/27.1.69, PMID: 14998240

[51] Chellappa SL Aeschbach D. Sleep and anxiety: from mechanisms to interventions. Sleep Med Rev. (2022) 61:101583. doi: 10.1016/j.smrv.2021.101583, PMID: 34979437

[52] Puzino K Frye SS LaGrotte CA Vgontzas AN Fernandez-Mendoza J. Am I (hyper)aroused or anxious? Clinical significance of pre-sleep somatic arousal in young adults. J Sleep Res. (2019) 28:e12829. doi: 10.1111/jsr.12829, PMID: 30714242

[53] Souissi A Farjallah MA Gaied Chortane O Dergaa I Mejri MA Gaamouri N. The effects of daytime melatonin ingestion on arousal and vigilance vanish after sub-maximal exercise: a pilot study. Eur Rev Med Pharmacol Sci. (2022) 26:6065–72. doi: 10.26355/eurrev_202209_29622, PMID: 36111906

[54] Hu S Liu X Wang Y Zhang R Wei S. Melatonin protects against body weight gain induced by sleep deprivation in mice. Physiol Behav. (2022) 257:113975. doi: 10.1016/j.physbeh.2022.113975, PMID: 36183851

[55] Ng KY Leong MK Liang H Paxinos G. Melatonin receptors: distribution in mammalian brain and their respective putative functions. Brain Struct Funct. (2017) 222:2921–39. doi: 10.1007/s00429-017-1439-6, PMID: 28478550

[56] Harris GC Aston-Jones G. Arousal and reward: a dichotomy in orexin function. Trends Neurosci. (2006) 29:571–7. doi: 10.1016/j.tins.2006.08.002, PMID: 16904760

