# Supplementary material for: Abnormal functional connectivity in the habenula is associated with subjective hyperarousal state in chronic insomnia disorder
Source: Front Neurol. 2023 Jul 31;14:1119595. doi: 10.3389/fneur.2023.1119595 (PMC10426801; doi:10.3389/fneur.2023.1119595)
Supplement: Supplementary file 1 [file Image_1.pdf]

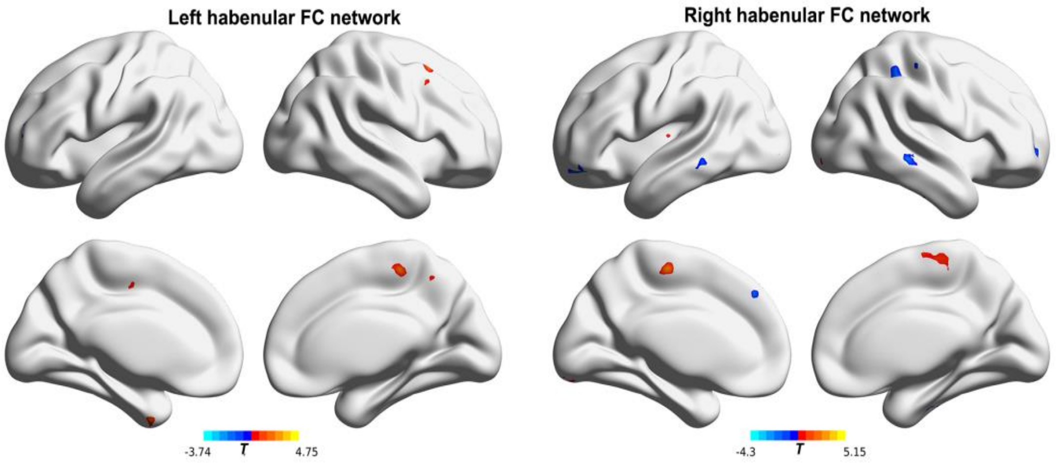

Figure S1. The group differences map in the bilateral habenular functional networks between CID patients with or without hyperarousal state. *p* < 0.05, uncorrected.
